# Epigenetic Mechanisms of Genomic Imprinting: Common Themes in the Regulation of Imprinted Regions in Mammals, Plants, and Insects

**DOI:** 10.1155/2012/585024

**Published:** 2012-02-15

**Authors:** William A. MacDonald

**Affiliations:** ^1^Department of Biology, Dalhousie University, Halifax, NS, Canada B3H 4R2; ^2^Departments of Biochemistry and Obstetrics & Gynecology, University of Western Ontario's Schulich School of Medicine and Dentistry, Children's Health Research Institute, London, ON, Canada N6C 2V5

## Abstract

Genomic imprinting is a form of epigenetic inheritance whereby the regulation of a gene or chromosomal region is dependent on the sex of the transmitting parent. During gametogenesis, imprinted regions of DNA are differentially marked in accordance to the sex of the parent, resulting in parent-specific expression. While mice are the primary research model used to study genomic imprinting, imprinted regions have been described in a broad variety of organisms, including other mammals, plants, and insects. Each of these organisms employs multiple, interrelated, epigenetic mechanisms to maintain parent-specific expression. While imprinted genes and imprint control regions are often species and locus-specific, the same suites of epigenetic mechanisms are often used to achieve imprinted expression. This review examines some examples of the epigenetic mechanisms responsible for genomic imprinting in mammals, plants, and insects.

## 1. Introduction

Epigenetic regulation of the genome is a critical facet of development. Epigenetic control of gene expression allows heritable changes in gene expression without the need for alterations in DNA sequence. This is achieved through the recruitment of molecular processes that assist transcription, block transcription, or degrade existing transcripts. Genomic imprinting is an epigenetic process that marks DNA in a sex-dependent manner, resulting in the differential expression of a gene depending on its parent of origin. Achieving an imprint requires establishing meiotically stable male and female imprints during gametogenesis and maintaining the imprinted state through DNA replication in the somatic cells of the embryo. Erasure of the preceding generation's imprint occurs in the germ line, followed by imprint reestablishment, in accordance with the sex of the organism. Each step in this imprinting process requires epigenetic marks to be interpreted by the genome and acted upon accordingly to result in parent-specific gene expression.

Genomic imprinting has been widely reported in eutherian mammals and marsupials [1–3]. Mice comprise the primary research model organism for the study of genomic imprinting. Approximately one hundred imprinted genes have been identified in mice with many more predicted to be present [2, 4]. This review considers imprinting to include chromosomal domains that direct imprinted epigenetic regulation, even if endogenous transcriptional units have yet to be identified as imprinting targets. Many imprinted genes in mice are developmentally important, linked to the formation of the placenta, or involved in brain function [2, 5, 6]. Noncoding transcriptional units, such as noncoding RNA, can also be imprinted and often form imprinted domains with developmentally important imprinted genes [7]. Imprinted genes found in mice are often used as candidates for investigating imprinted genes in other mammals. While some imprinted genes are conserved in mammals, many imprinted genes do not retain their imprinted status, even across eutherian mammals [1, 2]. For example, only a portion of the imprinted genes identified in mice are also known to be imprinted in humans [2], with placental-specific imprinted genes standing out in this discordance [8]. This demonstrates that imprinting cannot be predicted in nonmodel species simply by monitoring homologous genes. Additionally, this does not preclude the presence of imprinted genes or imprinted chromosomal regions being present in species outside of the existing documented examples. Determining how imprinting is lost in orthologous genes and what epigenetic changes are found within these regions can lead to a better understanding of how imprinted domains might be regulated.

In addition to mammals and marsupials, imprinted genes have also been identified in flowering plants [9, 10]. Imprinted chromosomes and chromosomal regions have been reported in insects [11], while transgenes have identified imprinted chromosomal regions in fish [12] and nematodes [13, 14]. Imprinted domains in chromosomal regions with unidentified target genes are seemingly dissociated from significantly influencing the development of these organisms, however, they are still subject to parent-specific epigenetic modifications and provide insight into the overall organization and mechanisms of genomic imprinting. While the function and characteristics of imprinted loci vary, both between and within organisms, there are some common themes of genomic imprinting. Many imprinted regions are either arranged in restrictive chromosomal areas or regulated as multigene clusters, indicating imprinted regions are contained as distinct structural domains. This organization may be related to the close association of imprinted domains to regions of the chromosome containing tandem repeats or transposable elements [9, 11, 15, 16]. It has further been suggested that these distinct imprinted domains could have a broader function to maintain genome integrity and assist in chromosome pairing, possibly contributing to the presence of such domains in diverse organisms [17].

 In this review, the epigenetic mechanisms involved in the regulation of imprinted domains in mammals, *Arabidopsis,* and *Drosophila* are explored. Mice represent the archetypal model for genomic imprinting and will be used to illustrate the differing roles of epigenetic mechanisms involved in regulating distinct imprinted domains. *Arabidopsis* is an emerging model organism for the study of genomic imprinting, where imprinting is pronounced in the endosperm but not the embryo proper. *Drosophila* are a model organism with a rich history in epigenetic research that have been utilized for transgenic imprinting element experiments while also having characterized imprinted chromosomal regions, despite not having any identified endogenously imprinted genes. Much remains to be understood about epigenetic regulation of genomic imprints. As epigenetic research expands to diverse model and nonmodel organisms, comparisons can be made between the structure and mechanisms of imprinted domains.

## 2. Common Epigenetic Mechanisms

The imprinted domains of mammals, plants, and insects represent distinct imprint events that do not share conserved sequence origins. While there are no universal templates that can be applied adequately to explain the regulation of all imprinted domains, either within or between organisms, there are common themes in the epigenetic mechanisms utilized and the multiple levels of regulation required to execute this parent-dependent mode of inheritance. As an epigenetic process, genomic imprinting alters gene expression without altering DNA sequence. However, DNA sequences are important in demarcating an imprinted domain. Imprinting control regions (ICRs) are often composed of repetitive DNA sequences found flanking, or internal to, imprinted genes, and in most cases, removal of an ICR will result in a loss of imprinting. Epigenetic modifiers of gene expression such as DNA methylation, histone modification, non-RNA, and higher-order chromatin formation act within ICRs to establish and maintain the imprinted state. ICRs act as nucleation sites for gene silencing or activation and are able to regulate expression of a single gene or an entire gene cluster. Enhancers and boundary elements are often associated with ICRs to restrict imprinted regulation to specific domains.

## 3. DNA Methylation

DNA methylation, the first epigenetic mechanism to be associated with imprinting, is an epigenetic modification that is applied directly to a strand of DNA [18, 19]. DNA methyltransferases (Dnmt) are highly conserved classes of enzymes that transfer methyl groups onto cytosine-C5 and are essential for both mammal and plant genome stability [20, 21], while being dispensable for the viability of *Drosophila*, which have low levels of genomic DNA methylation [22]. In plants and mammals, many ICRs contain differentially methylated regions (DMRs) that direct the epigenetic regulation of imprinted domains. Methylation within DMRs is often applied during gametogenesis and subsequently maintained throughout development, demonstrating the importance of DNA methylation for both the establishment and maintenance of many imprinted domains.

## 4. Histone Modification

Histone proteins and the modifications applied to them are highly conserved and comprise the most pervasive elements of imprinting across all taxa. Nuclear DNA is wrapped around nucleosomes, histone octamers composed of histones H2A, H2B, H3, and, H4, to form the basic repeating unit of chromatin. Various epigenetic modifications can be applied to the histones that affect chromatin conformation. Histone acetylation generally creates an accessible chromatin conformation while histone deacetylation, often coupled to histone methylation, initiates a compressed chromatin conformation that promotes silencing and the formation of heterochromatin [23]. Histone methylation can confer both an active or repressed transcriptional state depending upon which lysine is methylated. Histone 3 lysine 9 (H3K9), histone 4 lysine 20 (H4K20), and histone 3 lysine 27 (H3K27) are silencing modifications, while histone 3 lysine 4 (H3K4) methylation produces active chromatin [24]. Histone modifications and DNA methylation are often intertwined, each epigenetic mark can influence the other's recruitment to reinforce differential epigenetic states [25, 26]. Histone modifications at imprinted regions can also facilitate the formation of higher-order chromatin structures.

## 5. Higher-Order Chromatin Structures

Maintaining transcriptional inactivation of an imprinted allele often involves the formation of heterochromatin, a compacted chromatin structure that can spread in *cis* and generally impose transcriptional silencing. Heterochromatic regions remain stable throughout development and are propagated through cell division by late replication in S phase of the cell cycle [27]. Heterochromatic protein 1 (HP1) is a highly conserved nonhistone chromatin protein that is able to recruit other heterochromatic proteins and accessory factors, such as histone methyltransferases, to reinforce the structure of heterochromatin and initiate spreading in *cis *[28–30]. Polycomb group proteins form a silencing pathway largely parallel to heterochromatic silencing that targets homeotic genes [31]. Polycomb group silencing also involves histone deacetylases and histone methyltransferases, however, there is only modest overlap between Polycomb group and heterochromatic silencing.

## 6. Noncoding RNA, Antisense RNA, and RNA Interference

RNA interference (RNAi) is a highly conserved posttranscriptional silencing mechanism in which double-stranded RNA (dsRNA) are processed to form guides for the degredation of complementary RNA transcripts through an RNA silencing complex (RISC) [32, 33]. The production of noncoding RNA has been described at multiple imprinted regions in both mammals and plants [7, 34]. In many organisms, components of the RNAi silencing pathway are found to be involved in the recruitment DNA methyltransferases and other factors that facilitate higher-order chromatin structure [35]. As more imprinted domains in diverse organisms become characterized, noncoding RNA and RNAi may be found to have a significant role in the regulation of genomic imprinting.

## 7. Imprinting in Mammals

In mammals, most known imprinted genes are organized into clusters that share common ICRs to direct the parent-specific regulation of multiple genes within the cluster. Many mammalian ICRs contain differentially methylated regions (DMRs) that gain parent-specific DNA methylation marks either in the germline for imprint establishment, or in somatic cells for imprint maintenance. A survey of both human and mouse genomes found more tandem repeats in methylated regions of imprinted genes than methylated regions of nonimprinted genes [36]. The presence of these repeats may represent additional structural elements in imprinted regions that could direct chromatin alterations or recruit additional epigenetic mechanisms. The presence of noncoding RNA is another common feature of mammalian imprinting. In mice, extensive transcription of noncoding RNA has been reported at multiple imprinted loci, with many of these transcripts extending beyond the previously established boundaries of imprinted regions [37].

## 8. DNA Methylation and *Igf2-H19* Imprinting in Mammals

**Figure 1:**
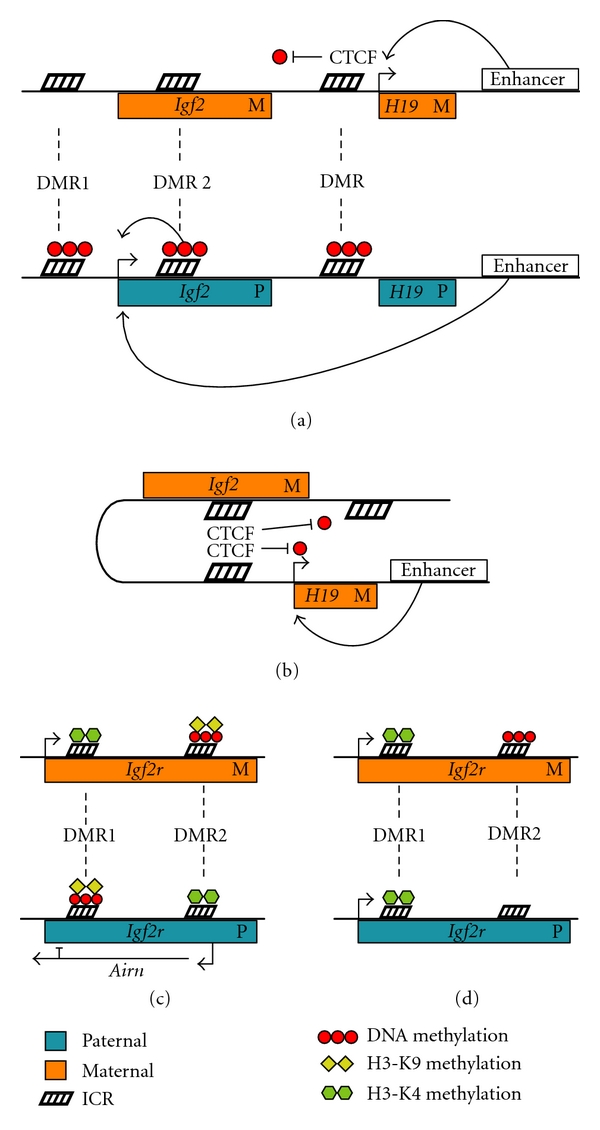
Imprinted regulation of *Igf2/H19* and *Igf2r/Airn *in mice and humans. (a) The *Igf2 *and* H19 *genes are reciprocally imprinted, with *H19* and *Igf2 *being expressed maternally and paternally, respectively. CTCF binds the maternal *H19* ICR and acts as an insulator sequestering enhancers to initiate maternal *H19* transcription while also protecting the *H19* ICR from methylation. Methylation on the paternal *H19* ICR prevents CTCF binding and silences paternal transcription. *Igf2* is only expressed paternally as a lack of CTCF binding in the paternal *H19* ICR allows enhancers to activate the *Igf2* promoter. DMR1 is a silencer that is inactivated by methylation while DMR2 is an enhancer that is activated by methylation. DMR1 and DMR2 are both methylated on the paternal allele, facilitating paternal *Igf2* transcription and blocking maternal transcription. (b) CTCF mediates an intrachromosomal loop, which prevents DNA methylation of the *H19* DMR and *Igf2 *DMRs, while facilitating *H19* expression. (c) In mice,* Igf2r* is maternally expressed while the overlapping *Airn *antisense transcript is paternally expressed. Histone H3K4 methylation in the maternal *Igf2r* promoter (DMR1) initiates transcription, while DNA methylation and histone H3K9 methylation in the downstream *Airn *promoter region (DMR2) silences maternal *Airn *transcription. Activating H3K4 methylation at the paternal *Airn *promoter region initiates paternal transcription of the *Airn *transcript. The *Airn *transcript overlaps the *Igf2r* promoter and contributes to the silencing of the paternal *Igf2r *allele along with DNA methylation and histone H3K9 methylation. (d) In humans, *Ifg2r* is biallelically expressed. Activating H3K4 methylation is found in both the maternal and paternal promoter regions of *Igf2r*. While maternal-specific DNA methylation of DMR2 is maintained, there is no H3K4 methylation of paternal DMR2, preventing the transcription of the *Airn *transcript.

The mouse *insulin-like growth factor 2* (*Igf2*) and *H19* genes were among the first imprinted genes to be characterized in detail [38, 39]. Subsequently, the same imprinting pattern was found for the human *Igf2* and *H19* genes [40, 41], leading to the imprinted status of *Igf2* becoming a standard assay for determining the presence of genomic imprinting in other vertebrates such as fish, birds, marsupials, sheep, and cattle [42–46]. The reciprocal imprinting of the* Igf2* and *H19* genes is mechanistically coupled. *H19* is maternally expressed and *Igf2* paternally expressed (Figure 1(a)). Two ICRs exist for *Igf2* and both are paternally methylated. DMR1, which is upstream of *Igf2* promoter 1, is a silencer that is inactivated by methylation [47]. DMR2 is located in exon 6 of *Igf2* and is an enhancer activated by methylation [48]. *H19* has one ICR which is located upstream of the *H19* gene and is also paternally methylated [49]. Regulation of the *Igf2 *and *H19* imprinted domains is dependent on paternal-specific DNA methylation within the DMRs to maintain monoallelic expression; deletions of the *H19* DMR and *Igf2* DMR1 or alterations to Dnmts result in biallelic expression of both *H19* and *Igf2* [50]. Passage through the germline is required to establish *Igf2*/*H19* DMR methylation [51], which is carried out by the Dnmt3a methyltransferase assisted by the Dnmt cofactor, Dnmt3L [52, 53]. Once established, paternal-specific methylation is then identified and maintained in somatic cells by Dnmt1 [54]. Dnmt1 cannot reestablish parent-specific DNA methylation patterns if prior methylation marks are lost [51].

 During mouse preimplantation development, both paternal and maternal genomes undergo extensive demethylation a few hours after fertilization. The paternal genome is demethylated rapidly by active demethylation while the maternal genome passively looses DNA methylation during each cell cycle [55, 56]. Imprinted DMRs must escape demethylation during preimplantation development to preserve any methylation marks established in the germline and this is achieved through the recruitment of maintenance methyltransferases to retain their methylated status [57]. In comparison to mice, sheep embryos have lower levels of genome reprogramming through preimplantation DNA demethylation [58], and only limited levels of active paternal genome demethylation [59]. An investigation into the epigenetic regulation of imprinted genes in sheep has found that parent-specific gene expression is not initiated until after the blastocyst stage, suggesting a later embryonic onset of parent-specific DNA methylation patterns [46]. Furthermore, *Igf2* and *H19* remain the only imprinted genes in sheep that have identifiable germline DMR methylation, the DMRs of other investigated imprinted genes only acquire parent-specific methylation marks later in embryonic development [46, 60]. Together, these results demonstrate that DNA methylation can be recruited to maintain silencing at imprinted regions that lack germline parent-specific DMRs, and that species-specific differences in genome regulation are reflected in the differential timing and recruitment of epigenetic mechanisms to maintain imprinted domains.

 The *Igf2* and *H19* imprinted domains remain one of the most studied examples of imprinting but much remains to be elucidated about the involvement DNA methylation at this imprinted domain. Ectopic localization of the *H19* DMR to a nonimprinted domain still results in paternal-specific DNA methylation of the DMR after fertilization despite the lack of germline establishment DNA methylation during spermatogenesis [61]. In order to achieve germline methylation of the ectopic *H19* DMR, additional DNA elements downstream of the endogenous *H19* DMR need to be included with the ectopic element [62]. These results suggest that more than DNA methylation alone is required to establish imprinting of this domain. Furthermore, in rare cases following DNA methylation disruption, a reversal of parent-specific imprinting patterns has been observed, including the H19 DMR gaining maternal DNA methylation and the paternal allele remaining unmethylated [63, 64]. These rare events may be due to the disruption of intrachromosomal connections or nuclear localization of the parental alleles. The DMRs of *Igf2* and *H19* can physically interact, potentially initiating parent-specific chromosome loops separating the two domains into active or repressed nuclear compartments [65]. Such separation of maternal and paternal alleles into different nuclear compartments may provide additional reinforcement for the maintenance of parent-specific expression [66, 67].

## 9. Chromatin Domains and the CTCF Insulator

The evolutionarily conserved CCCTC-binding factor (CTCF) is also involved in *Igf2* and *H19* imprinting. Within the *H19* ICR, there is a CCCTC binding site that is only functional on the maternal, unmethylated, allele. When CTCF binds the maternally unmethylated *H19* ICR, it acts as an insulator, blocking access of the *Igf2* promoter to enhancers [68]. Paternal methylation of the *H19* ICR inhibits CTCF binding, allowing enhancers access to the *Igf2* promoter on the paternal chromosome [69, 70]. Silencing of the *Igf2 *maternal allele is also facilitated by CTCF, which insulates maternal DMR1 and DMR2 from methylation when bound to the maternal *H19* ICR [71]. A loss of CTCF function results in *de novo *methylation of the maternal *H19* ICR, which effectively erases imprinted expression of *H19* and *Igf2 *[72]. Recent phylogenetic and mutational analysis has shown that the CTCF binding sites, and not DNA methylation of ICRs, are the more reliable predictor of the imprinted expression of *Igf2*. CTCF binding sites are conserved in humans, mice, and marsupials, which all have imprinted *Igf2 *and *H19*, while they are lacking in monotremes that do not imprint *Igf2 *or *H19 *[73]. Furthermore, *Igf2* DMR2 is biallelically methylated in both marsupials and monotremes, even though it is only biallelically expressed in monotremes, showing that methylation alone does not cause imprinted expression [73].

CTCF binds numerous sites within mammalian genomes, where it is identified both as a transcriptional regulator and a chromatin insulator able to block the spread of heterochromatin and mediated long-range chromosomal interactions [74]. CTCF-directed intrachromosomal loops are thought to contribute to parent-specific expression of *Igf2* and *H19 *(Figure 1(b)). Self-association between CTCF proteins bound to ICRs can initiate a chromosomal loop that isolates *H19* to maintain maternal expression, while reinforcing *Igf2* silencing through the creation of a repressive domain [75]. Disruption of CTCF binding to the maternal *H19* ICR results in *de novo* DNA methylation of maternal *Igf2* DMR1 and DMR2, suggesting that intrachromosomal looping mediates regulation of the entire maternal *Igf2*/*H19* imprinted region [76]. Isolation of imprinted alleles by CTCF has been reported at various other mammalian imprinted domains, where parent-specific binding of CTCF is critical for maintaining active expression from an imprinted allele [77]. However, it remains to be determined if the initiation of higher-order chromatin structures via CTCF-mediated intrachromosomal looping is a common feature of other imprinted domains.

## 10. Histone Modification and Mammalian Imprinting

Although DNA methylation has been the focus of the majority of studies on genomic imprinting in mammals, it is becoming clear that histone modification and RNA-based processes also play a critical role. The receptor of Igf2, *Igf2r*, is another well-characterized imprinted gene [78]. Rodents and marsupials imprint their* Igf2r* gene, while monotremes, birds, and primates (including humans) do not, and thus they have biallelic *Igf2r* expression [79]. In mice, *Igf2r* is maternally expressed, displaying a reciprocal pattern of imprinting to that of *Igf2* (Figure 1(c)). Two ICRs are present in *Igf2r*; the first, DMR1, is located in the *Igf2r *promoter region and is paternally methylated, and the second, DMR2, lies within the second intron of *Igf2r* and is maternally methylated. DMR2 corresponds to the promoter of an antisense RNA transcript *Airn *(formally *Air*), a large transcript that overlaps the promoter region of *Igf2r *[80]. The *Airn *transcript is exclusively paternally expressed and not only contributes to the silencing of paternal *Igf2r*, but also to the silencing of the genes which are in the same region as *Igf2r* yet do not overlap the *Airn* transcript [80].

 Histone methylation patterns are critical components of the parent-specific expression of *Igf2r *and* Airn *genes. In mice, the expressed maternal *Igf2r* allele and paternal *Airn *allele are both marked by H3K4 di- and trimethylation marks, while the repressed paternal *Igf2r* allele and maternal *Airn *allele are both marked by H3K9 trimethylation within the promoter region [81]. Indeed, histone methylation marks are more reflective of the imprinted state of* Igf2r* than the presence of *Airn *transcripts or DNA methylation patterns. In the mouse brain,* Igf2r* is biallelically expressed. This correlates with the presence of activating H3K4 methylation in both the paternal and maternal *Igf2r* DMR1 promoter region, despite the retention of paternal *Airn *transcription [81]. In humans, activating H3K4 methylation is present within both the maternal and paternal *Igf2r* promoter regions (Figure 1(d)) yet is absent from the *Airn *promoter region, eliminating *Airn *expression while facilitating biallelic *Igf2r* expression [81]. Recently, H3K4 demethylation is shown as a requirement for establishing imprinted silencing at some maternally repressed genes in mice, where the disruption of H3K4 demethylation prevented *de novo* DNA methylation of DMRs [82]. H3K4 demethylation appeared critical for imprinted genes that undergo *de novo* DNA methylation at later stages in embryonic development, suggesting the interaction between histone modifications and DNA methylation may be dependent on the developmental timing of epigenetic regulatory activity.

 A comprehensive survey of the histone modification present at imprinted regions compared to nonimprinted regions in mice determined three modifications closely associate with imprinted genes; repressed alleles contained H3K9 trimethylation and H4K20 trimethylation, while active alleles contained H3K4 trimethylation [83]. The chromatin state of imprinted regions was found to closely resemble heterochromatin and may be distinct from the general developmental silencing of genes, as H3K27 trimethylation was not present at all imprinted genes. The enrichment of H3K4, H3K9, and H4K20 trimethylation was present in imprinted genes regardless of whether the gene contained a DMR within its IRC, demonstrating both the importance and consistency of histone modification at imprinted domains. Broad enrichment of H3K27 trimethylation has been reported across some imprinted gene clusters [84]. This enrichment is occasionally biallelic and can be associated with both imprinted and nonimprinted genes alike within the same cluster [84]. Additionally, H3K27 trimethylation can also be disassociated from DNA methylation, or even antagonistic to DNA methylation within imprinted DMRs [85]. The complex association of H3K27 trimethylation with specific imprinted domains may be due to the secondary recruitment of H3K27 during development and tissue differentiation.

## 11. Antisense Transcripts and Mammalian Imprinting

The presence of noncoding RNA transcripts, such as the *H19 *and *Airn *RNAs, is associated with imprinted regions in mammals. Deletion of the DMR2 *Airn *promoter [86], or the truncation of the *Airn *transcript [80], results in paternal activation and biallelic expression of *Igf2r* and the neighboring gene clusters. Additionally, the *Airn *transcript is capable of maintaining paternal silencing in this gene cluster even if the paternal *Igf2r *promoter is experimentally activated [87] or if DNA methylation of DMR2 is lost [78]. Part of the silencing function of *Airn* may be the ability to recruit additional silencing complexes to the imprinted region. In the mouse placenta *Airn* can recruit the histone H3K9 methyltransferase G9a, which contributes to the imprinted silencing of the gene *Slc22a3 *within the* Igf2r *imprinted cluster [88]. Another important aspect of regulation by noncoding RNAs is the act of transcription itself and the interference such transcription can cause. It has been proposed that transcription of *Airn* through neighboring genes in *cis *contributes to their silencing [89]. Furthermore, the *Airn* transcript overlaps its own promoter and active transcription of *Airn* is required to prevent *de novo* methylation of this promoter on the paternal allele [90]. Recently, the transcriptional importance of noncoding RNAs been shown for the *Kcnq1* imprinted domain. In stem cells, targeted depletion of the *Kcnq1ot1* noncoding RNA did not relieve silencing of the paternally silenced genes, suggesting transcription through these genes during the production of *Kcnq1ot1 *contributes to their silencing more so than the presence of the *Kcnq1ot1 *transcript [91].

MicroRNAs (miRNAs) are endogenous 21–25 nt RNA transcripts that target complementary sequences for silencing [92]. Two miRNA genes, *miR-127* and *miR-136*, have been shown to be part of an imprinted domain responsible for the imprinted expression of the retrotransposon-like gene *Rtl1 *in mice and the orthologous *PEG11* gene in sheep and humans [93, 94]. Imprinted expression is associated with an unmethylated maternal ICR, leading to the miRNA genes only being maternally expressed which drives maternal-specific silencing of *Rtl1 *[95]. In sheep, *PEG11* produces a functional protein as well as an antisense *PEG11* transcript [96]. Imprinted silencing is directed by maternally produced antisense miRNA acting as guides for RISC-mediated destruction of maternal *PEG11 *transcript [97]. However, complex modulations of maternal miRNA generation suggest that maternal gene expression levels are balanced for dosage and not completely silenced [96, 97]. It is unclear if RNAi processing of *PEG11* transcripts by RNAi machinery recruits additional chromatic remodelers to regulate expression from the maternal allele.

 Genomic imprinting has been linked to dosage compensation in some mammals, where the silencing is directed towards the paternal  X chromosome [98]. In female mice, the paternal X chromosome is selectively silenced in extraembryonic tissues, in part by the production of the noncoding RNA *Xist*. Transcription of *Xist* spreads from an initial transcription site to cover most of the paternal X chromosome, leading to the recruitment of additional epigenetic silencing factors, such as histone methyltransferases and heterochromatic proteins [99]. Preferential silencing of the paternal  X chromosome still occurs if *Xist* noncoding RNA is lost, however, silencing is destabilized [100]. This may be related to the finding that the RNAi component Dicer is required for the spread of *Xist* and recruitment of the H3K27 trimethylation silencing in somatic cell X inactivation [101]. It is possible that imprinted silencing of the paternal X chromosome in extraembryonic mouse tissues originates from the imprinted silencing of specific target genes or regions, which then act as nucleation sites for RNAi-directed spreading of silencing across the whole chromosome.

## 12. Imprinting in Plants

Imprinting in plants was first documented in 1970, when it was found that a gene in maize produced fully colored kernels when maternally inherited and variegated kernels when paternally inherited [102]. In more recent years, genomic imprinting in angiosperms has been investigated extensively in *Arabidopsis*. Angiosperms experience double fertilization, with one sperm fusing the egg cell to produce the embryo proper, and the other fusing with the central cell to produce endosperm. The endosperm acts largely as support structure of the developing embryo and is terminally differentiated.

## 13. DNA Methylation in *Arabidopsis* FWA and FIS2 Imprinting

**Figure 2:**
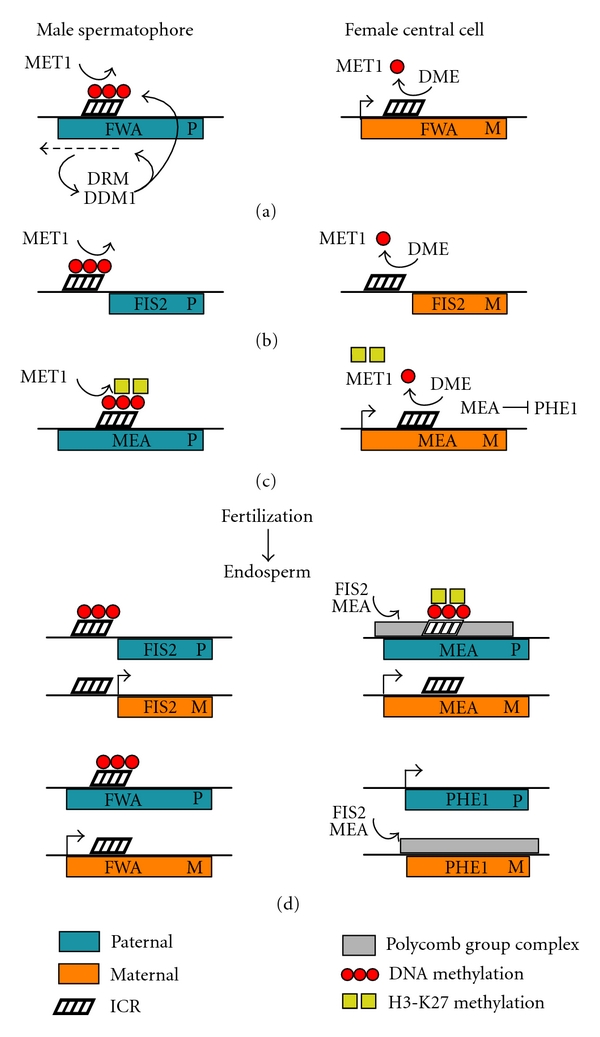
Imprinted regulation of the *Arabidopsis* genes FWA, FIS2, MEA, and PHE1. (a) Imprinted FWA is only expressed from the maternal allele. Prior to fertilization, MET1 methylates the paternal FWA promoter. In the male spermatophores, tandem repeats in the promoter produce siRNA (represented by the dashed arrow), which recruit DRM and DDM1 to the promoter region to maintain the methylated state. In the female central cell, DME demethylates the maternal FWA promoter maintaining maternal expression. (b) The antagonistic relationship between MET1 and DME is also involved in the imprinting of FIS2. MET1 methylates a region upstream of the paternal FIS2 allele that initiates silencing while DME demethylates the maternal allele. (c) The imprinted regulation of MEA also involves MET1 and DME; however, histone modification plays a key role in initiating parent-specific expression. Histone H3K27 methylation is present in the promoter region of the paternal MEA allele in addition to DNA methylation. DME protects the maternal promoter from both DNA and histone methylation. Transcribed maternal MEA, which encodes a member of the Polycomb group silencing complex, initiates the parent-specific silencing of maternal PHE1. (d) In the endosperm, the Polycomb group gene MEA contributes to its own imprinted expression in the endosperm, with maternally produced MEA involved in the silencing of the paternal MEA allele. FIS2, which is also part of a Polycomb silencing complex, contributes to silencing the paternal MEA allele. PHE1, which is regulated by Polycomb group silencing, is only expressed from the paternal allele. Maternally produced FIS2 and MEA combine to maintain the silencing of the maternal PHE1 allele.

The* Arabidopsis* gene FWA encodes a homeodomain-containing transcription factor involved in the regulation of flowering and is a well-characterized imprinted gene expressed solely from the maternal allele [103]. FWA imprinting involves DEMETER (DME), a DNA glycosylase able to excise modified nucleotide bases and the MET1 methyltransferase (Figure 2(a)). MET1 methylates tandem repeats in the FWA promoter and DME acts to remove methylated cytosines from the maternal FWA allele, leaving only the paternal FWA allele methylated [103, 104]. If DME demethylation is lost, the imprint is also lost, as both maternal and paternal FWA alleles remain methylated by MET1 [103, 104]. This scenario implies methylation is the default state and active demethylation is required to imprint an allele. DME is primarily expressed in the female central cell before fertilization and is not expressed until long after fertilization or in the male sporophyte [105]. This disparity in DME expression provides a window during which the imprint can be established on the maternal FWA allele prior to fertilization but requires additional mechanisms to maintain expression after fertilization. FWA, FERTILIZATION INDEPENDENT SEED 2 (FIS2) is also maternally expressed and is regulated through the antagonistic action of DME and MET1 (Figure 2(b)). A distinct 200 bp region upstream from FIS2 acts as the nucleation center for FIS2 paternal methylation but, unlike the MET1 methylation site in the FWA gene, there are no tandem repeats in this region [106]. For both FWA and FIS2, active MET1 methylation is required during male gametogenesis to produce paternal-specific silencing [106].

## 14. RNAi and Heterochromatin Formation in *Arabidopsis* FWA Imprinting

RNA-directed DNA methylation (RdDM) is a process that produces locus-specific heterochromatin formation in angiosperms and is attributed to the need to silence transposons. Initially, dsRNA is processed by RNAi machinery into small interfering RNAs (siRNA). These siRNA then guide site-specific DNA methylation and heterochromatinization [107]. Methylation produced by RdDM does not spread significantly in *cis* so silencing is precisely targeted to the region producing the dsRNA [108]. Heterochromatin formation arising from the RdDM pathway involves the ATPase chromatin-remodeling factor DECREASE IN DNA METHYLATION1 (DDM1), an SWI/SNF homologue involved with the maintenance of H3K9 histone methylation and DNA methylation [107].

 The FWA promoter contains tandem repeats that produce dsRNA from the paternal FWA allele, which guides DDM1 methylation and heterochromatin formation [107]. The function of DDM1 is exclusively in the maintenance of silencing as FWA methylation cannot be reestablished by DDM1 after siRNA or DNA methylation is lost [109]. Mutations in genes involved in the RNAi pathway of *Arabidopsis*, including *dicer-like3* and *argonaute4*, result in a loss of paternal FWA methylation It has been proposed that the siRNA generated from the FWA promoter tandem repeats also guides DOMAINS REARRANGED METHYLTRANSFERASE (DRM), a Dmnt3 homologue, to perform *de novo* methylation [110]. This shows that the RNAi pathway in *Arabidopsis* can initiate silencing of targeted imprinted domains.

## 15. Histone and Polycomb Group Proteins in *Arabidopsis* Imprinting

The *Arabidopsis* Polycomb group protein MEDEA (MEA) gene is imprinted, resulting in expression exclusively from the maternal allele in the endosperm (Figure 2(c)). Similar to FWA and FIS2 imprinting, MEA regulation also involves DME activation and MET1 DNA methylation [111]. However, while DNA methylation is found in the promoter region of the paternal MEA allele, it likely does not play a large role in the initial regulation of the imprint [112]. Transcriptional activation of maternal MEA is maintained in the female central cell by DME [105], while the paternal MEA allele is silenced by H3K27 histone methylation [106]. Paternal MEA silencing is maintained by a Polycomb group complex, which includes FERTILIZATION INDEPENDENT ENDOSPERM (FIE), FIS2 and the maternally produced MEA [106, 113]. This Polycomb group complex is able to initiate a self-reinforcing loop of silencing, maintaining H3K27 methylation and recruiting additional Polycomb complexes.

 MEA not only assists in regulating its own imprinted expression but also causes a cascade of imprinted expression in the genes that it regulates. The gene PHERES1 (PHE1) is regulated by the imprinted MEA protein and, as a consequence, is also imprinted [114]. PHE1 encodes a type I MADS-box protein, a protein family typically involved in DNA binding, and leads to uncontrolled endosperm proliferation when overexpressed. MEA, acting as part of a multiprotein complex with other Polycomb group proteins, forms condensed chromatin structures at its binding site within the PHE1 promoter which silences the PHE1 gene (Figure 2(d)) [115]. As only the maternal MEA allele is active prior to fertilization in the endosperm, PHE1 Polycomb silencing is also limited to the maternal allele [114]. The imprinting of both MEA and PHE1 demonstrates that the imprinting of a regulatory gene can produce a cascade of parent-specific gene expression. Recently, the gene *Phf17* (*Jade1*), which encodes for a component of the HBO1 histone 4 acetylation complex, has been found to be imprinted in the mouse placenta [116]. This finding is interesting as it suggests the possibility of similar downstream imprinting events in the mouse placenta as those found in *Arabidopsis* endosperm.

## 16. The *mee1* Gene Is Imprinted in the Maize Embryo

While all imprinted genes in *Arabidopsis* have so far been found to be monoallelically expressed only in the endosperm, a gene in maize, *maternally expressed in embryo 1 *(*mee1*), is reported to have parent-specific expression in both the endosperm and embryo [117]. Maternal-specific expression of *mee1* in the endosperm is regulated in a manner similar to that described for *Arabidopsis,* with maternal-specific active DNA demethylation and protection from DNA methyltransferases. The paternal *mee1* allele is methylated in gametes and remains methylated at all stages of development, preventing paternal transcription. The maternal allele is also methylated DNA in gametes; however, active demethylation of a DMR located near the transcriptional start site of *mee1* occurs after fertilization, suggesting that the initial parent-specific demarcation of the alleles is independent of DNA methylation. During gamete production, the maternal allele regains DNA methylation within the DMR. It remains to be determined which epigenetic mark establishes the maternal imprint but, it appears as though the *mee1* DMR is in fact a differentially demethylated region, which may be a reflection of species-specific epigenetic reprogramming dynamics. Regardless, this finding illustrates the ability of the maize genome to maintain parent-specific demarcation of genes in the developing embryo, and predicts the identification of further genes with imprinted embryonic expression in plants.

## 17. Imprinting in Insects

The investigation of imprinting in insects has progressed quietly since early studies in *Sciara* and *Coccids* revealed that gene silencing induced by whole chromosome heterochromatinization was dependent on the parental origin of the chromosome [118, 119]. It was the study of chromosome elimination in the fungus gnat, *Sciara,* which leads to the use of the descriptive term “imprint” [120]. Crouse reported that X chromosomes acquire an “imprint” which directs paternally derived X chromosomes to be eliminated from somatic cells and ensures that only the female X chromosomes remain in the gametes [120]. This work provided explicit evidence of parent-specific silencing. Whole chromosome imprinted regulation such as this is not uncommon in insects [121]; however, parent-specific transcriptional silencing of smaller chromosome regions, similar to that found in mammals and plants, has also been described in *Drosophila*.

## 18. Genomic Imprinting in *Drosophila*

Thus far, all imprinted domains in* Drosophila melanogaster* have been found only in chromosome regions that are heterochromatic [11]. In *Drosophila*, most heterochromatin is compartmentalized into large blocks such as those flanking the centromeres, the entire Y chromosome, and in a few discrete regions that are developmentally controlled. The relegation of imprinted domains to gene poor chromosomal regions is advantageous as it limits parent-specific silencing to relatively few genes [122]. This property also has made identifying endogenous imprinted genes in *Drosophila* difficult as these regions are mostly uncharacterized. Most known imprinted domains in *Drosophila* have been detected through position-effect variegation (PEV), which causes variegated transcriptional silencing of gene clusters placed adjacent to heterochromatic regions. Using transgenes or reporter genes placed into heterochromatic regions, imprinted domains have been identified by the display of parent-specific PEV silencing of the marker gene. The majority of the *Drosophila* Y chromosome is imprinted, as inserted transgenes are silenced in a parent-specific manner [123, 124], while distinct imprinted domains have been reported in heterochromatic regions of the X chromosome and the autosomes [11, 125, 126].

## 19. Imprinting of the *Drosophila Dp(1;f)LJ9* Mini-X Chromosome

**Figure 3:**
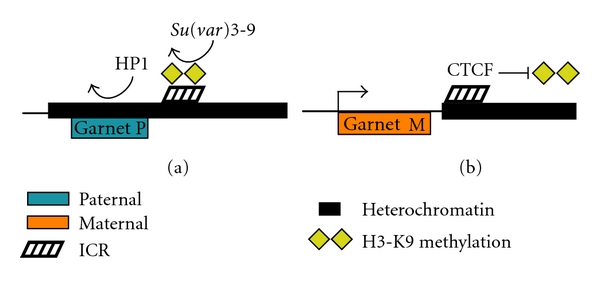
Creation of the* Drosophila* mini-X chromosome and the resulting imprinted expression of the garnet marker gene. The *Dp(1:f)LJ9* mini-X chromosome was generated through an inversion followed by a large deletion by X-ray irradiation. In the resulting mini-X chromosome, *garnet* is placed next to a region centric of heterochromatin containing an imprinting center. (a) Paternal transmission of the mini-X chromosome results in silencing of *garnet*, as a result of H3K9 methylation and heterochromatin formation. (b) Maternal transmission of the mini-X chromosome results in active transcription of the *garnet* gene, maintained by CTCF counteracting heterochromatin formation.

The *Drosophila Dp(1:f)LJ9* mini-X chromosome is the result of an X chromosome inversion and deletion which juxtaposes euchromatic genes to a heterochromatic *Drosophila* imprinting center [126, 127]. One of the euchromatic genes that falls under control from the imprinting center is the eye color gene *garnet. *This gene is uniformly expressed when maternally inherited and exhibits variegated silencing when paternally inherited, and so acts as a reporter for the imprint. Mutations which alter PEV by either enhanced silencing (*E(var*)) or suppressed silencing (*Su(var)*) do so by affecting proteins and accessory factors involved in heterochromatin formation. An extensive screen of the effects of *Su(var)* mutations on imprinted* garnet* expression revealed that both HP1 (*Su(var)2–5*) and the H3K9 histone methyltransferase (*Su(var)3–9*) were required for the maintenance of the paternal imprint (Figure 3(a)) [128]. Additionally, a mutation of *Su(var)3-3*, responsible for H3K4 demethylation [129], also disrupted the silencing of the paternally inherited *Dp(1:f)LJ9 *[128]. This suggests active removal of the activating H3K4 methylation mark is required before H3K9 methylation can direct HP1 recruitment and the formation of heterochromatin. While Polycomb group proteins have been implicated in the regulation of both mammalian and plant imprinting [6, 130], they do not appear to have any role in epigenetic regulation from the *Dp(1:f)LJ9 *imprinting center. Mutations in Polycomb group genes, including *Enhancer of zeste E(z)* which initiates H3K27 methylation, have no effect on paternal-specific silencing [128].

 None of the *Su(var)* mutations tested on *Dp(1:f)LJ9 *had any effect on the stability of the maternal imprint, demonstrating that maternal inheritance of *Dp(1:f)LJ9* allows a stable boundary to form between the marker gene and the ICR to counteract heterochromatinization. The compact *Drosophila* genome utilizes many insulator proteins to create regulatory domains, but only the CTCF insulator protein is highly conserved [131, 132]. Similar to the role of CTCF in maintaining mammalian imprinted domains, CTCF also acts to protect maternally inherited *Dp(1:f)LJ9 *by acting as a boundary element against the spread of heterochromatin (Figure 3(b)) [133]. Other insulator proteins remain to be fully tested for their involvement in the *Dp(1:f)LJ9* maternal-specific boundary, however, Suppressor of Hairy-wing (Su(Hw)) and the *Drosophila*-specific Boundary Element-associated Factor (BEAF-32) do not appear to be necessary [134]. In *Drosophila,* many non-CTCF insulator proteins depend on PcG and Trx group proteins for proper function [135]. The failure of PcG and Trx group mutations to modify maternal *Dp(1:f)LJ9 garnet* expression [128] suggests non-CTCF insulators are not likely to be recruited to the maternal boundary. The specific involvement of CTCF with the *Dp(1:f)LJ9* imprint is intriguing as it raises the possibility that the imprint was acquired prior to *Drosophila* speciation or that the factors contributing imprint maintenance are more likely to involve conserved epigenetic mechanisms.

 The role of heterochromatin at the *Dp(1:f)LJ9* imprint center is limited to imprint maintenance; no *Su(var) *mutations, Polycomb group protein mutations, or chemical heterochromatin modifiers impacted either the maternal or paternal establishment of the imprint [126, 128]. Similarly, CTCF is not involved in establishment of the maternal imprint [133], mirroring of its role in mammalian imprinting where it is also dispensable for imprint establishment [68, 136]. These findings illustrate the fact that distinct epigenetic mechanisms are used for the establishment and maintenance of parent-specific expression from the *Dp(1:f)LJ9* ICR. Establishment of the imprint requires correct passage through the germline, as evidenced by the loss of the *Dp(1;f)LJ9* paternal imprint in cloned *Drosophila *[137].

 Regulation of the *Dp(1;f)LJ9* imprinting center demonstrates features of both discrete mammalian ICRs and whole chromosome imprinting characteristics found in other insects. Paternal inheritance of the disrupted imprinting region results in the spreading of heterochromatic silencing to proximal areas; a similar spreading of silencing from an imprinted region has also been described in mammals [138]. However, a secondary effect of the exposed paternal *Dp(1;f)LJ9* ICR is a chromosome-wide decrease in transcription, similar to the imprinted silencing of whole chromosomes in *Coccids *[122]. The stable maternal boundary generated from the *Dp(1;f)LJ9* ICR prevents both the local spreading of heterochromatin and the chromosome-wide reduction of transcription [122]. This finding suggests that silencing initiated from a heterochromatic imprinted domain is able to impose long-range *cis* alterations in regulation when not properly insulated within a heterochromatic region.

## 20. Noncoding RNA and Imprinting in *Drosophila*

*Drosophila* dosage compensation involves an increase in male X chromosome expression instead of the silencing of one female X chromosome, as occurs in mammals [139]. Increased transcription of the male X chromosome coincides with the binding of the male-specific lethal (MSL) complex, which is recruited to specific chromosome sites by the noncoding RNAs *roX1* and *roX2* [139]. Deletion of both *roX* genes eliminates compensated expression from genes on the X chromosome, resulting in male lethality [140]. Similar to the stabilization role of *Xist* in spreading of X chromosome silencing in mice, the MSL complex is still able to colocalize to specific X chromosome sites and direct limited activation in the absence of roX [139]. The spreading of MSL transcriptional activation, however, is dependent on *roX* RNA transcription [141]. Recently, it has been reported that experimental manipulation causing maternal inheritance of the Y chromosome significantly relieves male lethality caused by *roX* mutations, suggesting imprinted regions on the Y chromosome augment *roX* expression [142]. This suggests that correct passage of the Y chromosome through the male germline results in the establishment of epigenetic marks that influence dosage compensation in *Drosophila*. It has been proposed that the imprinted regions of the Y chromosome may contribute to hybrid incompatibility between *Drosophila *species [142], a phenomenon previously associated with imprinted genes in both mammals and plants [143, 144].

## 21. DNA Methylation and Imprinting in Insects

There is a precedent for the involvement of DNA methylation in insect imprinting in the mealybug *Planococcus citri*. Complete silencing of paternally inherited chromosomes in males is associated with DNA hypomethylation [145]. In this case, hypomethylated chromosomes, which have been inherited paternally, become silenced in males, while chromosomes inherited maternally remain hypermethylated and active. The epigenetic imprint marking paternal chromosomes for silencing appears to be H3K9 di- and trimethylation, which is established during gametogenesis, while the lack of H3K9 di- and trimethylation on the maternal chromosomes may simply reflect a default imprinted state [146]. Heterochromatic spreading reinforces the silent state of paternal chromosomes, as HP1-like and HP2-like complexes are recruited to chromosomes with H3K9 di- and trimethylated histones [147]. It is proposed that silencing of entire paternal chromosomes is nucleated from discrete ICRs marked by H3K9 di- and trimethylation, which escape early embryonic activation signals, and propagates chromosomal silencing [146]. Such spreading of silencing, originating from discrete ICRs to cover the entire chromosome, corresponds to the mechanisms guiding parent-specific chromosomal regulation described in *Drosophila* and mouse extraembryonic tissues.

*Drosophila* possess a single DNA methyltransferases, Dnmt2, and only have low genome-wide levels of DNA methylation that peak early in embryogenesis and decline towards adulthood [22, 148]. The presence of DNA methylation in the developing embryo is defined developmentally, as nuclear concentrations peak in the early embryo then begin to decline as development progresses [22, 149]. *Drosophila* with *Dnmt2* mutations remain fertile and viable with no observable phenotype [22], however, overall lifespan is diminished [150]. Recently, Dnmt2 has been implicated in the genomic regulation of retrotransposons, suppressing retrotransposon transcription in somatic cells of the early embryo [151]. Loss of Dnmt2 resulted in the mislocalization of the H4K20 methyltransferase, resulting in the elimination of H4K20 trimethylation and reduced retrotransposon repression. Dnmt2 was also shown to be associated with heterochromatin formation at repeat transgene arrays, illustrating the potential for DNA methylation to assist in the recruitment and stabilization of heterochromatic factors in *Drosophila *[151].

 The role of Dnmt2 in retrotransposon repression does not extend to the germline [151]. This finding is supported by research involving transgenic *Drosophila* with mammalian Dnmts; flies overexpressing mammalian Dnmts are not viable [152], however, germline-specific expression of mammalian Dnmts does not effect fertility or the viability of progeny [153]. Together, these findings suggest that genomic regulation by DNA methylation in *Drosophila* is restricted to somatic cells, and unlike mammals and plants, does not have an essential role in in the germline. While the role of DNA methylation in *Drosophila* development is still an area of great debate [154, 155], current research would suggest that DNA methylation is not a candidate for a germline establishment epigenetic mark in *Drosophila* imprinting.

## 22. Recognition of Mammalian Imprinting Elements in Transgenic *Drosophila*

Various transgenic *Drosophila* lines have been produced that contain either mouse or human ICRs [156–158]. These ICRs function as silencers in *Drosophila* but do not confer parent-specific silencing. Similar experiments involving human ICRs introduced into transgenic mice also resulted in a loss of parent-specific regulation [159, 160]. Transgenic studies involving the mouse *H19* ICR exemplify remarkable conservation of epigenetic function between the mouse and *Drosophila* genomes. A specific region of the upstream *H19* ICR was identified as a silencing element in mice by first being identified as a required sequence for silencing in transgenic *Drosophila* [161]. Furthermore, the production of noncoding RNA transcripts from the upstream *H19 *ICR was also first discovered in transgenic *Drosophila*, where noncoding RNA production from the transgenic insert was associated with reporter gene silencing [162]. The upstream *H19 *ICR is necessary for proper repression of paternal *H19 *expression in mice [163], where the noncoding transcripts are thought to be involved in the recruitment of other silencing mechanisms [162]. Both of these studies involving the transgenic mouse *H19* ICR identified endogenous silencing mechanisms using a transgenic system, demonstrating the potential for epigenetic regulatory fidelity between two distinct organisms.

 The *Drosophila* insulator Su(Hw) and Polycomb group proteins, Enhancer of zeste (E(z)) and Posterior sex combs (Psc), were found to regulate the transgenic *Igf2/H19* ICR construct [164]. These results show that imprinted transgenes are able to recruit histone modifiers and chromatin remodelers to direct silencing of a chromosomal domain. The binding of Su(Hw) to the transgenic *Igf2/H19* ICR construct is reminiscent of CTCF binding to the endogenous *H19* ICR in mice [68]. In mice, CTCF protects *H19 *from methylation and silencing, whereas in *Drosophila* Su(Hw), binding to the *H19* ICR initiates downstream silencing, possibly by the recruitment of heterochromatic factors. The involvement of Su(Hw) with silencing from the *H19* ICR is specific to this imprinted element. Typically, Su(Hw) protects transgenes from silencing in *Drosophila* [165] and other ICRs are not dependent on Su(Hw) for silencing in transgenic *Drosophila* [164]. This unexpected involvement of Su(Hw) with the *H19 *ICR suggests that elements within the ICR are eliciting a genomic response from *Drosophila* that are beyond that of a nondescript repetitive element.

 An intriguing finding from the mammal-*Drosophila* transgenic imprinting experiments is that silencing activity is often maintained, but the insulator/boundary activity necessary for maintaining gene expression is lost. Expression from an imprinted domain requires the parent-specific recruitment of both silencing and activating chromatin remodelers, which includes insulators. Binding of *Su(Hw)* to the transgenic *H19* ICR did not produce the same insulator properties as endogenous CTCF binding provides, but, rather, acted as a silencer [164]. Furthermore, multiple transgenic constructs, produced from sections of both human and mouse *H19* ICRs, all acted as silencing elements in *Drosophila* but did not retain any of their insulator functions [166]. These findings could suggest that the maintenance of the active component of imprinted regions might be equally as complex as the silenced component and may require species-specific recognition of epigenetic marks. The complexity of imprinted large domains and their association with repressed repetitive elements could favor robust regulatory mechanisms to ensure the maintenance of active imprinted alleles, as exemplified by the complex intrachromosomal folding associated with maternal activation of *H19* (Figure 1(b)). Together, these transgenic experiments show that while many epigenetic mechanisms utilized for silencing genes are highly conserved, the elements that superimpose the parental specificity of silencing are more specialized and tailored to the regulatory needs of each species.

## 23. Common Epigenetic Mechanisms Regulate Diverse Imprinted Domains

Producing parent-specific expression requires independent regulation of the maternal and paternal alleles. Histone modification and DNA methylation, leading to heterochromatin formation, are common regulators of imprinted silencing. Noncoding RNA and RNAi are emerging as critical components for the early recruitment of silencing mechanisms to ICRs. Boundary elements have also been shown to be necessary to maintain discrete regulatory domains by protecting active alleles, in a parent-specific manner, from silencing by blocking either the recruitment or spreading of silencing mechanisms. In all cases, genomic imprinting relies on multiple epigenetic mechanisms acting in concert to maintain and reinforce silencing.

 The recent identification of H3K4, H3K9, and H4K20 trimethylation as an epigenetic marks common to imprinted genes in mice is a significant step in understanding the epigenetic code that constitutes the demarcation of a genomic imprint [83]. As high-throughput screening of genome-wide epigenetic modifications is explored in more organisms, it will be interesting to see if a similar, concise pattern of epigenetic modifications emerges. In *Drosophila*, both H3K9 and H3K4 methylation are associated with the *Dp(1;f)LJ9* imprinted domain, while H3K27 methylation is not [128]. The finding that H3K27 trimethylation was found at some, but not all, imprinted genes in mice [83], yet is the primary histone modification associated with imprinting in *Arabidopsis*, may reflect the role of H3K27 trimethylation as a ubiquitous epigenetic modification in *Arabidopsis* [167]. This highlights that species-specific variations in the use of epigenetic regulators such as DNA methylation or RNAi will be reflected in how an imprinted region is regulated. Variation in the structure of an imprinted domain, and the organism in which it found, will result in differential reliance on specific epigenetic mechanisms and, possibly, the order in which they are recruited. Evolutionary pressures and the species-specific arrangement of chromosomes also factor into the construction of large imprinted domains or novel genes acquiring imprinted regulation. Nevertheless, in all species examined here, common suites of epigenetic processes appear to be employed to regulate genomic imprinting.

 The study of genomic imprinting has progressed for the better part of a century but it is still very much in its infancy. Complex regulatory patterns continue to be revealed within known imprinted regions and new imprinted genes continue to be discovered. Assessing imprinting in diverse model and nonmodel organisms can broaden the understanding of what epigenetic processes are necessary to achieve an imprint. Despite the fact that specific imprinted genes are not often conserved between diverse species, the epigenetic mechanisms and gross structural features of imprinted regions are often similar. Recognizing the common processes of genomic imprinting will aid our understanding of the epigenetic mechanisms required to distinguish maternal and paternal genomes during development in both model and nonmodel organisms.
